# Editorial: Insights into the mechanisms of transcranial electrical stimulation

**DOI:** 10.3389/fnins.2023.1286239

**Published:** 2023-09-18

**Authors:** Elias Manjarrez, Gianluca Campana, Claudio Mirasso, Lorella Battelli

**Affiliations:** ^1^Instituto de Fisiología, Benemérita Universidad Autónoma de Puebla, Puebla, Mexico; ^2^Department of General Psychology, University of Padova, Padua, Italy; ^3^Instituto de Física Interdisciplinar y Sistemas Complejos (IFISC, UIB-CSIC), Palma de Mallorca, Spain; ^4^Department of Neurology, Berenson-Allen Center for Noninvasive Brain Stimulation, Beth Israel Deaconess Medical Center and Harvard Medical School, Boston, MA, United States; ^5^Center for Neuroscience and Cognitive Systems@UniTn, Istituto Italiano di Tecnologia, Rovereto, Italy

**Keywords:** Transcranial Electrical Stimulation (TES), transcranial Alternating Current Stimulation (tACS), Transcranial Random Noise Stimulation (tRNS), transcranial Direct Current Stimulation (tDCS), mechanisms of action (MoA)

Transcranial Electrical Stimulation (TES) (Nitsche and Paulus, 2000, 2001; Roche et al., 2015; Bikson et al., 2018, 2019; Antal et al., 2022) has been now extensively validated as a promising technique for non-invasive neuromodulation of cortical functioning in the healthy population, with exciting potential applications in treating various neurological disorders. This Research Topic sheds light on the intricate physiological mechanisms of action that underlie diverse TES modalities alone or in combination, such as transcranial Direct Current Stimulation (tDCS), transcranial Alternating Current Stimulation (tACS), alternating current galvanic vestibular stimulation (AC-GVS), and Transcranial Random Noise Stimulation (tRNS) applied on the cerebral cortex or the cerebellum (Figure 1). By harnessing experimental and computational methods, and the simultaneous application of TES on multisite brain regions, researchers are unraveling the multifaceted effects of these techniques on brain activity and function. The following description of cutting-edge studies offers a glimpse into the diverse insights gained from this collection of research.

**Figure 1:**
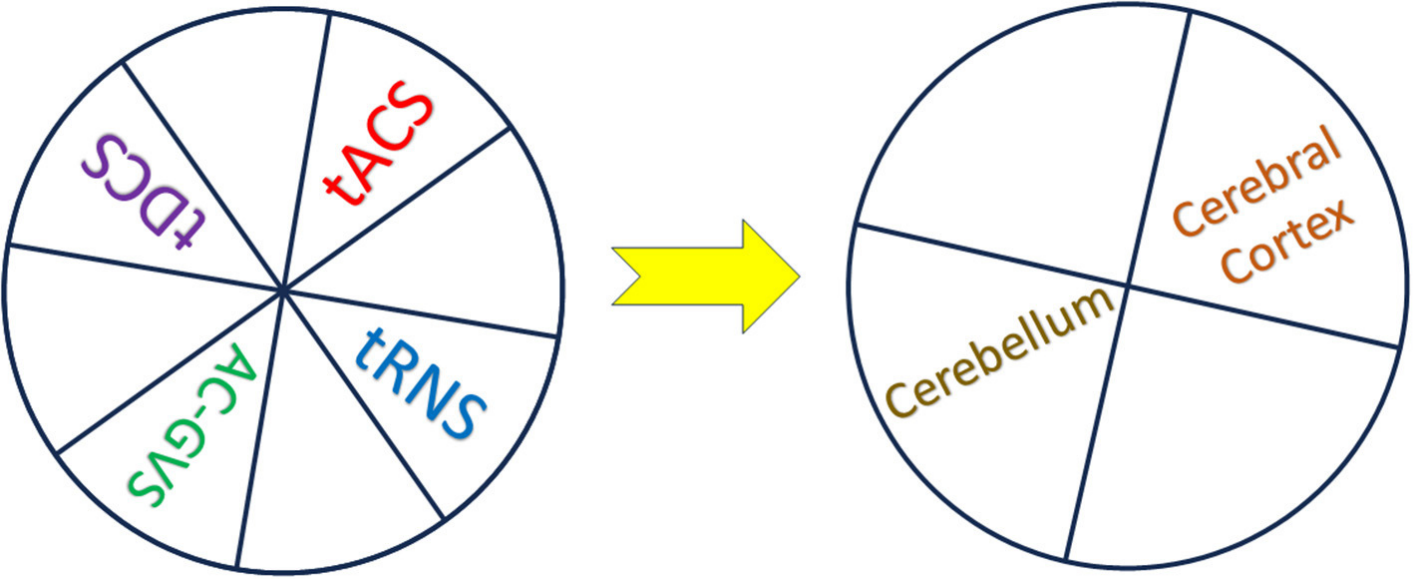
In this Research Topic, Transcranial Electrical Stimulation (TES) modalities alone or in combination were applied to multisite brain regions at the cerebral cortex and cerebellum: transcranial Direct Current Stimulation (tDCS), transcranial Alternating Current Stimulation (tACS), Alternating Current Galvanic Vestibular Stimulation (AC-GVS), and Transcranial Random Noise Stimulation (tRNS). Experimental and computational methods were combined to highlight the multifaceted effects of these techniques on brain activity and function.

Cabrera-Álvarez et al. embarked on a journey to better comprehend the effects of tACS on cortical gyrification. They applied individual alpha frequency (IAF)-tACS over parieto-occipital (PO) areas to increase alpha power and analyze the current distribution with computational modeling, taking into account the impact of individual gyrification. Computational modeling and experimental results converged, revealing enhanced alpha power across various brain regions. The experimental results aligned with prior research, revealing enhanced alpha power of about 8% across multiple brain regions, except for specific PO areas (Kasten et al., 2019; see also Zaehle et al., 2010; Helfrich et al., 2014; Zarubin et al., 2020), whereby the computational modeling revealed that the distribution of normal electric field components in parietal regions was different. Specifically, the researchers found that the key predictor of alpha power increase in the PO areas was the average of the electric field's normal components. Surprisingly, gyrification's impact was 2-fold, exerting opposing hyper-/depolarization effects in distinct brain areas, not directly targeted by tACS. This study could help understand the intricate mechanics that drive tACS-induced brain activity changes. As experimental neuroscientists venture toward harnessing non-invasive brain stimulation for clinical applications, this research highlights the need for further computational exploration to exploit their potential for treating brain disorders effectively. This study is relevant because it could help optimize the use of non-invasive brain stimulation in clinical settings, in which tACS has shown promise in restoring disrupted alpha rhythms by synchronizing with the individual alpha frequency (Lustenberger et al., 2015; Grabner et al., 2018).

Nakazono et al. embarked on an innovative journey by coupling two non-invasive brain stimulation techniques: tACS over the cerebellum (Koganemaru et al., 2020) and alternating current galvanic vestibular stimulation (AC-GVS) (Coats, 1972). Participants were required to mentally simulate walking from a first-person perspective while standing in a relaxed position with closed eyes. Inertial measurement unit sensors were placed on different body parts, including the head, upper thoracic regions, pelvis, and feet, to measure yaw, pitch, and roll angles. The authors unveiled an imagery-driven phenomenon linked to in-phase stimulation, exhibiting an effect on yaw peak power. Their results reveal insights associated with the potential of a phase-dependent modulation within the vestibular-cerebellar network, elucidating the mechanism behind the observed effects. This unique approach, combining tACS and AC-GVS is a significant step toward advancing therapeutic applications, pioneering an innovative protocol of bi-transcranial neurostimulation research.

Qurat-ul-ain et al. described that in stroke rehabilitation, tDCS has primarily been used on the cerebral motor cortex (Salameh et al., 2022), although more recently, on the cerebellum (Mohammadi et al., 2021; Solanki et al., 2021). Their study shows a detailed comparison of anodal tDCS in these two regions (see also Ehsani et al., 2016). In a meticulously designed trial, stroke patients underwent anodal tDCS (or sham control) over the motor cortex or the cerebellum during gait and balance training. The striking results revealed that the stimulation of the motor cortex or the cerebellum significantly improved gait and balance, with comparable effects. This research unveils a versatile aspect of tDCS, spotlighting its potential to safely be combined with other therapies, and more importantly, the potential of targeting different brain regions to yield comparable rehabilitation outcomes.

Herzog et al. comprehensively analyzed transcranial current stimulation methods, specifically focusing on the cerebellum (Spampinato et al., 2021). By employing a range of excitability measures, including motor evoked potential (MEP) amplitude and cerebellar brain inhibition (CBI), the study aimed at identifying the most effective stimulation method to boost the cortical response. The results unveiled intriguing dynamics: while both tACS and tRNS increased MEP amplitude, tDCS showed no effect. However, the study did not show any impact on sensor-based movement analysis or other inhibitory measures. The research underscores the importance of further investigations to elucidate the intricate mechanisms underlying the efficacy of these stimulation methods.

As the readers can note, this Research Topic highlights a deeper understanding of TES mechanisms in different brain regions. By merging empirical studies of multisite TES with computational modeling, researchers visualize the potential therapeutic benefits of TES methods for neurological disorders. As we delve into this field, the findings are promising in augmenting brain dynamics, cognition, and clinical outcomes via non-invasive brain stimulation.

## Author contributions

EM: Conceptualization, Supervision, Validation, Writing—original draft, Writing—review and editing. GC: Supervision, Writing—review and editing. CM: Supervision, Writing—review and editing. LB: Supervision, Writing—review and editing.
